# Depression clinical trials worldwide: a systematic analysis of the ICTRP and comparison with ClinicalTrials.gov

**DOI:** 10.1038/s41398-024-03031-6

**Published:** 2024-07-31

**Authors:** Eugenia D. Namiot, Diana Smirnovová, Aleksandr V. Sokolov, Vladimir N. Chubarev, Vadim V. Tarasov, Helgi B. Schiöth

**Affiliations:** 1 Department of Surgical Science, Functional Pharmacology and Neuroscience, University of Uppsala, Uppsala, Sweden; 2 Advanced Molecular Technologies, Limited Liability Company (LLC), Moscow, Russia

**Keywords:** Depression, Clinical pharmacology

## Abstract

Major depressive disorder (MDD), commonly known as depression, affects over 300 million people worldwide as of 2018 and presents a wide range of clinical symptoms. The international clinical trials registry platform (ICTRP) introduced by WHO includes aggregated data from ClinicalTrials.gov and 17 other national registers, making it the largest clinical trial platform. Here we analysed data in ICTRP with the aim of providing comprehensive insights into clinical trials on depression. Applying a novel hidden duplicate identification method, 10,606 depression trials were identified in ICTRP, with ANZCTR being the largest non- ClinicalTrials.gov database at 1031 trials, followed by IRCT with 576 trials, ISRCTN with 501 trials, CHiCTR with 489 trials, and EUCTR with 351 trials. The top four most studied drugs, ketamine, sertraline, duloxetine, and fluoxetine, were consistent in both groups, but ClinicalTrials.gov had more trials for each drug compared to the non-ClinicalTrials.gov group. Out of 9229 interventional trials, 663 unique agents were identified, including approved drugs (74.5%), investigational drugs (23.2%), withdrawn drugs (1.8%), nutraceuticals (0.3%), and illicit substances (0.2%). Both ClinicalTrials.gov and non-ClinicalTrials.gov databases revealed that the largest categories were antidepressive agents (1172 in ClinicalTrials.gov and 659 in non-ClinicalTrials.gov) and nutrients, amino acids, and chemical elements (250 in ClinicalTrials.gov and 659 in non-ClinicalTrials.gov), indicating a focus on alternative treatments involving dietary supplements and nutrients. Additionally, 26 investigational antidepressive agents targeting 16 different drug targets were identified, with buprenorphine (opioid agonist), saredutant (NK2 antagonist), and seltorexant (OX2 antagonist) being the most frequently studied. This analysis addresses 40 approved drugs for depression treatment including new drug classes like GABA modulators and NMDA antagonists that are offering new prospects for treating MDD, including drug-resistant depression and postpartum depression subtypes.

## Introduction

Major depressive disorder (MDD), commonly known as depression, is a severe medical condition characterized by a wide range of clinical symptoms. As of 2018, the number of people suffering from depression surpassed 300 million worldwide [1]. Surveys conducted in 30 countries estimated the lifetime prevalence of depression to be approximately 10.8% [2]. A recent study has indicated a significant increase of 27.6% in new MDD cases, affecting over 53 million individuals, largely attributed to the COVID-19 pandemic [3]. Among patients diagnosed with MDD, approximately one-third do not respond to a combination of antidepressants, resulting in higher rates of comorbidity and unfavorable outcomes [4]. Despite extensive research efforts, the underlying pathophysiology of depression still remains elusive, hindering the development of novel medications.

One of the most recognized hypotheses associates depression with the depletion of serotonin and noradrenaline, leading to the development of medications aimed at modifying the levels of these neurotransmitters [5]. The most commonly prescribed groups of drugs for depression are Selective Serotonin Reuptake Inhibitors (SSRIs) and Serotonin Noradrenaline Reuptake Inhibitors (SNRIs) [6, 7]. However, it has been reported that around 30% of patients with MDD do not respond to these medications, and less than 60% achieve remission [8, 9]. Alternative hypotheses propose that the depletion of these neurotransmitters is a consequence of depression development, possibly due to chronic inflammation [8]. A systematic review involving over 13,000 depressed patients revealed that more than a quarter of them displayed low-grade inflammation and elevated levels of C-reactive protein [10]. MDD patients have also shown increased levels of IL-6, Il-1b, and TNF-a, indicating the potential for anti-inflammatory medications to be effective [8, 11, 12]. An increasing number of clinical trials have reported improved treatment outcomes with several anti-inflammatory drugs, including NSAIDs, immunodepressants, cytokine inhibitors, glucocorticoids, statins, antibiotics (such as minocycline), and even certain stimulants (like modafinil) [13–17]. However, due to the heterogeneity of anti-inflammatory drugs and the high risk of side effects, many studies emphasize the importance of identifying specific groups of individuals who are suitable candidates for this treatment [13, 16].

The “leaky gut” hypothesis suggests that inflammation and the build-up of neurotoxic substances, such as quinolinic acid [9], may be linked. Recent studies have shown that the gut becomes more permeable, allowing lipopolysaccharide (LPS) from gram-negative enterobacteria to enter the bloodstream, triggering cytokine production [18, 19]. In randomized placebo-controlled clinical trials, an 8-week probiotic regimen led to a reduction in the Beck Depression Index and lower levels of C-reactive protein [20, 21]. Even shorter periods of probiotic intake,

like 30 days of *Lactobacillus helveticus* and *Bifidobacterium longum*, have shown to lower stress levels [22]. Another potential benefit of probiotics is their ability to reduce depression risks among healthy individuals [23]. However, some reviews suggest that most clinical trials haven’t shown significant results, especially in people over 65 years old [23, 24]. It’s essential to further explore these findings and their implications.

Depression has been linked to disturbances in corticotropin-releasing hormone (CRH) levels [25]. Chronic stress often triggers heightened cortisol release, a phenomenon supported by other studies [26]. Moreover, hypercortisolism is associated with an increased production of pro-inflammatory cytokines, which in turn can diminish serotonin levels via the kynurenine pathway and decrease crucial neurotrophic factors [27]. Consequently, this lays the groundwork for the utilization of medications such as metyrapone, mifepristone, and fludrocortisone, all of which target the hypothalamic-pituitary-adrenal (HPA) axis. While trials have shown limited efficacy of fludrocortisone due to the necessity for higher doses, which can result in hypertension, metyrapone has demonstrated promise as an adjunct to antidepressant therapy [28]. Mifepristone, functioning as a competitive glucocorticoid receptor antagonist, is primarily under investigation for its potential in treating psychotic depression [29, 30]. In various psychiatric and neurodegenerative conditions, patients have shown specific polymorphisms in the FKBP5 gene, which plays a role in negative feedback regulation [31–33]. Consequently, individuals with mutations in this gene experience reduced sensitivity of glucocorticoid receptors, leading to uncontrolled CRH release [25, 34]. To address this issue, CRH1 antagonists were proposed as potential solutions. Unfortunately, despite extensive trials, these drugs showed no significant changes in any stress-related disorder, which has hindered their further use [35–38]. However, researchers are now exploring the modulation of the glucocorticoid system with FKBP5 antagonists, which has demonstrated promising results in preclinical studies [39, 40].

Despite the emergence of new depression treatments, the literature highlights the prevalence of drugs targeting serotonin, noradrenaline, and dopamine signaling [41–44]. Moreover, SSRIs and SNRIs were found to possess additional anti-inflammatory properties. When combined with other drugs like folate, they can achieve improved clinical outcomes [42, 44]. One possible option is vortioxetine, a novel serotonin transport blocker, which has demonstrated anti- depressive efficacy comparable to duloxetine and venlafaxine [45]. In a comprehensive review involving 8 547 patients from several clinical trials, vortioxetine exhibited favorable clinical outcomes and better tolerability than SNRIs [46]. Additionally, certain drugs commonly used in other medical areas, such as ketamine, have shown a robust antidepressant effect [47].

The diversity in causes and mechanisms of depression presents numerous potential new therapeutic targets and significant improvements to existing treatments. Combining information from different databases, such as the WHO International Clinical Trials Registry Platform ICTRP and ClinicalTrials.gov [48–50] may provide a better comprehensive understanding of the available treatment options. Surprisingly, no analytical reviews to date have evaluated clinical trials of drugs for depression using the ICTRP. The existing review papers on clinical trials of depression treatments show considerable heterogeneity. Many articles primarily focus on evaluating psychotherapy and other non-invasive therapy methods rather than the administration

of specific drugs [51, 52]. On the other hand, pharmacotherapy reviews often concentrate on a particular drug/pharmacological class or subtype of Major Depressive Disorder (MDD), such as treatment-resistant MDD [53–55]. The purpose of this analysis is to shed light on the recent global trends in depression treatments, using the two largest clinical trials databases: the ICTRP and ClinicalTrials.gov databases.

## Material and methods

### Data acquisition

We obtained comprehensive data from the ICTRP platform and imported it into a PostgreSQL 9.6 relational database, timestamped on May 24, 2021. This dataset encompasses clinical trials registered between 1990 and 2020 and includes information such as trial and secondary IDs, scientific and public titles, study type, study design, phase, registration date, enrollment date, target size, recruitment status, primary sponsor, countries involved, conditions studied, interventions, and bridged type. In addition to the clinical trial data, we incorporated datasets from Medical Subject Headings (MeSH, 2021 version), the National Library of Medicine (NIH) drug datasets, and Drug Bank datasets into our relational database. Moreover, we utilized the R programming language for statistical computation and analysis purposes as previously described [56–59].

To categorize the clinical trials based on their conditions, we employed the MeSH (Medical Subject Headings) database. This comprehensive database contains common entry terms and categories for various medical conditions. We merged the MeSH database’s categories with the Conditions in the ICTRP dataset, creating a new Condition table that associates conditions with each trial’s ID. We focused on specific MeSH categories, namely Diseases[C], Behavior and Behavior Mechanisms [F01], and Mental Disorders [F03], as they were most relevant to diseases. Other non-relevant categories were excluded from the dataset. For drug categorization, we used drug names, categories, and possible synonyms from the Drug Information Portal of the National Library of Medicine (NIH). We matched all conditions with their synonyms to identify hidden duplicates effectively. To compile a list of approved and investigational drugs used in depression trials, we used DrugBank. Within DrugBank, we analyzed datasets using the R package dbparser, extracting essential data points such as drugbank_id, type, name, and synonym.

### Data pre-processing

Data preparation is a critical step in any data analysis, significantly impacting the quality of the results. As a fundamental rule, the quality of the output is directly linked to the quality of the input data. We began by eliminating all entries lacking a registration date. Trials without registration dates were considered incomplete and therefore excluded from our analysis. Duplicate entries in the ICTRP platform can arise when a single clinical trial is registered independently through multiple registries. These duplicates can be categorized into two types: visible duplicates, which are flagged and resolved by the platform itself, and hidden duplicates,

which exist in the system without clear marking. Our data preparation involved identifying and addressing both types of duplicates to ensure the accuracy of our analysis. To address visible duplications, we leveraged system flags, particularly the “Bridged_type” field, which allowed us to easily identify and remove these duplicates.

Handling hidden duplicates involved a more complex process, as described in a previous publication [56]. In this process we standardized the gender values within secondary identifiers. This involved separating values using semicolons and removing country suffixes for identifiers originating from the European Clinical Trials Register. This normalization facilitated the selection of records with matching secondary identifiers. We identified potential hidden duplicates by comparing records with the same target size and primary sponsor. We also assessed the similarity between combined public and scientific trial names and checked for matching names in the NIH drug dataset. The similarity score for combined titles was calculated using the Levenshtein distance algorithm. From these potential duplicates, we retained the trial with the earliest enrollment date and, when enrollment dates were identical, the trial with the earliest trial ID in alphabetical order.

We also changed some field values. Diseases falling under more than one category were assigned to one primary category. Matching drugs could be classified into one or more groups: approved, illegal, recalled, research, experimental, nutraceutical, or veterinarian-approved. If a drug was approved anywhere, it was considered approved. If the drug was recalled at some point, it was classified as withdrawn. Study and experimental drugs did not differ. The drugs were also classified using the NIH drug categories.

Several test phases were replaced with the earliest mentioned phase. The recruitment status was categorized as completed when the word “complete” was mentioned in the recruitment status column. For recruitment status of “recruiting”, “open public recruiting”, “open to recruitment”, “enrolling by invitation”, “authorised-recruitment may be ongoing or finished”, “ongoing”, “approved for marketing”, “not yet recruiting” or “active, not recruiting”, it was categorized as “Active”. A trial was categorized as “Not Active” if recruitment status contained “terminate”, “withdrawn”, “stopped”, “suspended”, “pending”, “not recruiting”, “closed to recruitment of participant”. Trials with a missing or not done recruitment status were categorized as unknown.

For a more accurate analysis, we tried to leave as many clinical trials as possible in the output set. MeSH database entry terms were used to merge with clinical trial conditions. Depression trials were filtered using the following grid terms: depressive disorder (F03.600.300) and depression (F01.145.126.350; F01.470.282). Trials that were not aggregated to this exact category were scanned using the following query terms (obvious misspellings included): “depression” OR “depresion” OR “depressive” OR “depresive”.

### Data analysis and approved drugs

We filter records where the “drugbank_id” is not empty and has a length greater than 3. This step helps us eliminate incorrect entries (those with invalid “drugbank_id” values). The records in the database are not normalized. Therefore, in the first pass of the “interventions” field, we accumulate information about all available “drugbank_id,” “drugbank_type,” “drugbank_group,” and “drugbank_name” values, which are extracted from the “name” field. In the second pass, multiple assignments from the “interventions” field are expanded into separate records. We distinguish between Clinical Trials and Non-Clinical Trials based on the presence of the “NCT” prefix in the “trialId” field. The domain value in the “url” field is used to determine the country of registration. We filtered out records where the values in the “drug_name” field clearly did not fit the definition of “drugs,” such as “water,” “watermelon,” and so on, up to a count of 20. Subsequently, we determine the top 30 drugs by grouping records based on the “drugbank_name” field.

To find the approved drugs, we web-scraped https://nctr-crs.fda.gov/fdalabel/ui/search using the following terms in the ―Labelling Full Text Search‖ field: ―Major Depressive Disorder‖, ―Treatment resistant depression‖ and ―Depression‖. Attained lists of approved drugs were then extended using DrugBank website. Some drugs were detailed for the lack of approvement by either Food and Drug Administration (FDA) or European Medicines Agency (EMA). Investigational indications were found using clinicaltrials.gov.

## Results

### Depression trials characteristics

We delved into the characteristics of depression trials and conducted a comparison between different registries. In total, we identified 10 606 depression trials, with the majority being interventional trials, totaling 9229. We compared various aspects, including the phase of the trials, target size range, recruitment status, allocation, masking, and the use of drug treatment. Upon analysis, we found that there were 5261 trials registered on ClinicalTrials.gov and 3968 on non-ClinicalTrials.gov registries. Interestingly, in both cases, the majority of trials had not specified the phase (58.9% for ClinicalTrials.gov and 74.5% for non-ClinicalTrials.gov trials). Notably, ClinicalTrials.gov had a higher percentage of trials reaching phase 4 (11%) compared to non-ClinicalTrials.gov, which only had 4.4% of trials in phase 4. Conversely, a significant proportion of non-ClinicalTrials.gov trials were in phase 2 (7.9%). Regarding the trial status, most of the ClinicalTrials.gov trials were completed (3061, 58.2%), while non-ClinicalTrials.gov had only 45% of trials completed, with 35.4% of trials still active. Notably, non-ClinicalTrials.gov displayed a larger percentage of trials that were not active, with 740 non-ClinicalTrials.gov trials versus 430 ClinicalTrials.gov trials falling into this category.

**Fig. 1:**
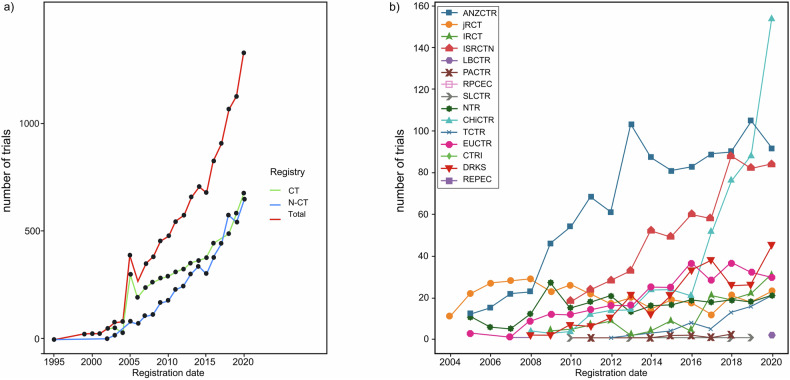
Annual numbers of registered trials between registries. **a** When considering this comparison, ClinicalTrials.gov consistently outpaced non-ClinicalTrials.gov databases in most years. However, an interesting anomaly occurred in 2018 when non-ClinicalTrials.gov databases reached their peak with 501 registered trials, while ClinicalTrials.gov reported 484 registrations. Notably, ClinicalTrials.gov experienced a remarkable peak in 2005. **b** Taking a closer look at individual non-ClinicalTrials.gov registries, we found that ANZCTR led the pack with the highest number of clinical trials, totaling 1 031. ANZCTR exhibited two peaks, one in 2013 and another in 2019. Other prominent databases included IRCT with 576 trials and ISRCTN with 501 trials. Despite CHiCTR’s total of 489 trials, it displayed an exceptional growth pattern, registering the highest number of trials in a single year, reaching 160 trials. CT ClinicalTrials.gov; N-CT non-ClinicalTrials.gov, ACTRN Australian New Zealand Clinical Trials Registry, CHiCTR Chinese Clinical Trial Registry, CTRI Clinical Trials Registry - India, DRKS German Clinical Trials Register, EUCTR, EU Clinical Trials Register, IRCTN Iranian Registry of Clinical Trials, ISRCTN International Standard Randomized Controlled Trial Number Registry, JPRN Japan Primary Registries Network, NTR The Netherlands National Trial Register, PACTR Pan African Clinical Trial Registry.

We then performed a study on the yearly number of clinical trials across various registries. Out of a total of 614 208 trials analyzed, depression trials accounted for 1.7% (n = 10,606). Figure 1 illustrates the trend lines depicting the number of registered trials per year from 1995 to 2020. Notably, the annual number of trials (Fig. 1A) displayed a significant increase from 2004 onwards, with a peak observed in 2005, where 308 studies were registered on ClinicalTrials.gov. Furthermore, our findings revealed a noteworthy shift in 2018, as the number of non-ClinicalTrials.gov trials surpassed those registered on ClinicalTrials.gov for the first time. Specifically, 484 trials were registered on ClinicalTrials.gov, while non-ClinicalTrials.gov registries accounted for 501 trials. This observation indicates a growing involvement of non-ClinicalTrials.gov platforms in depression research.

We then conducted a detailed study of non-ClinicalTrials.gov databases (Fig. 1B). Among these databases, Australian New Zealand Clinical Trials Registry (ANZCTR) emerged as the largest repository with 1031 depression trials. ANZCTR displayed an upward trend with two prominent peaks in the years 2013 and 2019. Following ANZCTR, the other top four databases included Iranian Registry of Clinical Trials (IRCT) with 576 trials, International Standard Randomised Controlled Trial Number (ISRCTN) with 501 trials, Chinese Clinical Trial Register (CHiCTR) with 489 trials, and The EU Clinical Trials Register (EUCTR) with 351 trials. The most striking observation was the significant growth of depression trials in the CHiCTR database after the year 2016. In fact, by 2020, CHiCTR had the highest number of registered clinical trials, almost reaching 160. Similarly, the ISRCTN database stood out with nearly 100 new trials registered per year since 2017. On the other hand, all other non-ClinicalTrials.gov databases did not exceed 50 trials per year throughout the entire period from 1995 to 2020. These findings highlight the changing landscape of depression research, with ANZCTR, CHiCTR, and ISRCTN playing increasingly significant roles in hosting a substantial number of clinical trials related to depression.

### Pharmacotherapy for depression

**Fig. 2:**
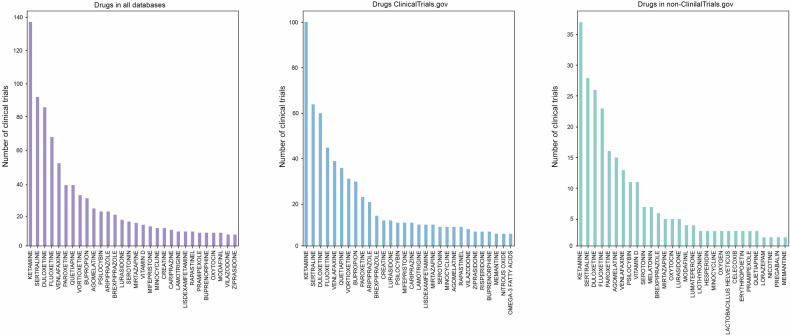
Drugs in ClinicalTrials.gov and non-ClinicalTrials.gov. Among the drugs studied in clinical trials, ketamine stands out as the most extensively researched, with 100 trials listed in ClinicalTrials.gov and 37 in non-ClinicalTrials.gov sources. Additionally, three other drugs that garnered significant attention were sertraline, duloxetine, and fluoxetine. ClinicalTrials.gov generally hosted more studies for each drug. Venlafaxine was another notable drug, making it to the top 10 drugs in both ClinicalTrials.gov and non- ClinicalTrials.gov listings. However, in the non-ClinicalTrials.gov category, venlafaxine was surpassed by agomelatine (with 15 trials) and paroxetine (with 16 trials). Notably, some less common substances like *Lactobacillus Helveticus*, minocycline, modafinil, celecoxib, erythropoietin, and hesperidin made appearances in the non-ClinicalTrials.gov group, but each of these drugs had fewer than 5 trials associated with them. In contrast, in the ClinicalTrials.gov group, we found such intriguing drugs as minocycline, mifepristone, and psilocybin, each with fewer than 20 trials. It’s worth highlighting that a novel NMDA receptor modulator called rapastinel was exclusively found among the top drugs listed on ClinicalTrials.gov.

The DrugBank database was utilized to analyze drugs studied in both ClinicalTrials.gov and non-ClinicalTrials.gov databases (Fig. 2). To analyze the most prominent drugs, we developed a string similarity algorithm using the Levenshtein distance. Interestingly, the top four most studied drugs were the same in both groups, with ketamine being the most extensively studied drug, followed by sertraline, duloxetine, and fluoxetine. However, ClinicalTrials.gov showed a larger number of trials dedicated to each drug compared to the non-ClinicalTrials.gov group. For example, in the ClinicalTrials.gov group, ketamine was present in 100 trials, while the non-ClinicalTrials.gov group had only 37 trials involving the drug. Similarly, venlafaxine played a significant role in the ClinicalTrials.gov group, with almost 40 trials, but in the non-ClinicalTrials.gov group, it was outnumbered by agomelatine (15 trials) and paroxetine (16 trials). In the non-ClinicalTrials.gov group, we also noted the presence of other drugs and probiotics such as modafinil, minocycline, *Lactobacillus helveticus*, celecoxib, and erythropoietin, each with less than 5 trials dedicated to them. In the ClinicalTrials.gov database, psilocybin, mifepristone, lisdexamfetamine, creatine, nitrous oxide, and omega-3 fatty acids were present in less than 20 trials each. Overall, all databases displayed similar drugs with a slight shift towards non-regular antidepressant therapy, as evidenced by the inclusion of substances like oxytocin and nicotine in the non-ClinicalTrials.gov group. This suggests a potential exploration of alternative or adjunctive treatment approaches beyond traditional antidepressant medications in the non-ClinicalTrials.gov trials.

**Fig. 3:**
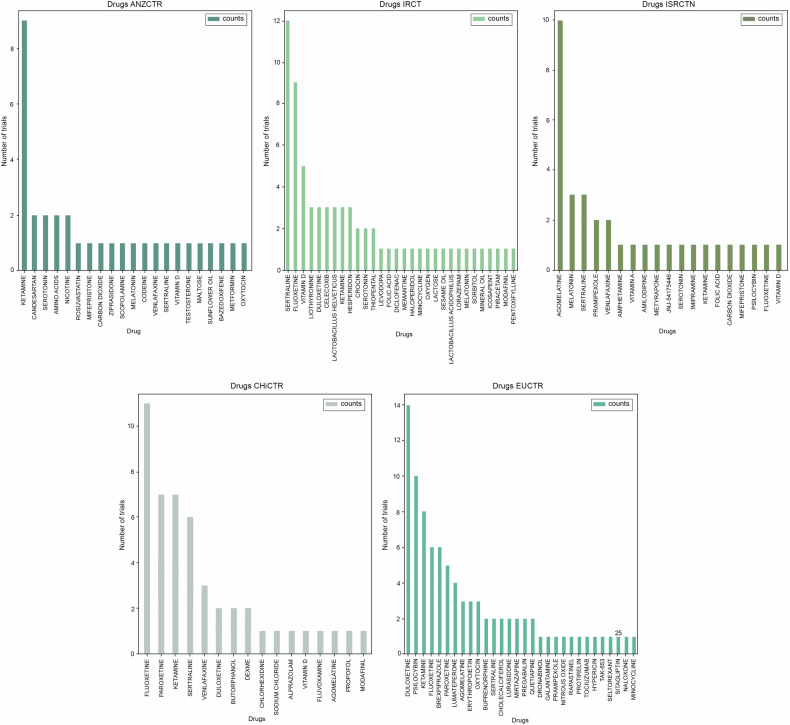
Drugs in the non-ClinicalTrials.gov group. Upon closer examination of the non-ClinicalTrials.gov group, we identified five databases with the largest number of clinical trials: ANZCTR (978 trials), IRCT (576 trials), ISRCT (501 trials), CHiCTR (489 trials), and EUCTR (351 trials). While the top drugs differed among these databases, it’s noteworthy that ketamine, sertraline, duloxetine, fluoxetine, and venlafaxine consistently ranked in the top 10 drugs in all five databases. The presence of unique drugs in each database highlights the importance of analyzing each database to gain a comprehensive understanding. For example, the ANZCTR database featured unexpected entries such as nicotine, amino acids, codeine, and even sunflower oil in clinical trials. The IRCT database exclusively highlighted *Lactobacillus Helveticus* as one of the top studied drugs. ISRCTN notably focused on agomelatine, with nearly 10 trials dedicated to this drug, which differed significantly from the CHiCTR database where agomelatine appeared in only 1 trial. CHiCTR, in turn, emphasized drugs like fluoxetine, paroxetine, and ketamine. Despite these variations, it’s important to note that in all databases, the overall number of clinical trials for each drug did not exceed 15 trials, indicating that the research landscape is characterized by a wide array of drugs being studied in relatively small numbers. The EUCTR database stood out for its relatively higher concentration of trials centered around specific drugs, including 14 trials on duloxetine, 10 trials on psilocybin, and 8 trials on ketamine. Additionally, two experimental drugs, TAK-653 (present in the EUCTR, 1 trial) and JNJ-54175446 (in ISRCTN, 1 trial), were identified.

Among the non-ClinicalTrials.gov databases, we identified the top 5 databases based on the number of trials they contained: ANZCTR (978 trials), IRCT (576 trials), ISRCTN (501 trials), CHiCTR (489 trials), and EUCTR (351 trials) (Fig. 3). It is important to mention that in almost all cases, the most popular drugs did not exceed a total of 15 trials. Furthermore, the top drugs varied across each database. However, we observed that ketamine, sertraline, duloxetine, fluoxetine, and venlafaxine consistently appeared in the top 10 drugs in all five databases. Compared to the other databases, ANZCTR stood out for its inclusion of nicotine (2 trials), amino acids (2 trials), codeine (1 trial), and sunflower oil (1 trial). However, it is essential to mention that these substances were only present in a maximum of two trials each, indicating that their usage in clinical trials is relatively limited. Moreover, during our analysis, we discovered that the IRCT database exclusively featured *Lactobacillus helveticus* in three trials and acidophilus in one trial. Moreover, minocycline was exclusively present in the top drugs of IRCT and EUCTR. The IRCT database also exhibited several anti-inflammatory drugs, such as celecoxib (3 trials) and diclofenac (1 trial).

In the ISRCTN database, the atypical antidepressant agomelatine stood out significantly, surpassing all other drugs with a total of 10 trials. Additionally, we came across drugs like amphetamine, amlodipine, metyrapone, and mifepristone, each present in only one trial. On the other hand, the CHiCTR database seemed to primarily focus on fluoxetine (11 trials), paroxetine (7 trials), ketamine (7 trials), sertraline (6 trials), and venlafaxine (3 trials). Notably, unlike ISRCTN, agomelatine appeared in only one trial in CHiCTR. It is worth mentioning the presence of dexamethasone and modafinil in CHiCTR, accounting for 2 trials and 1 trial, respectively. In the EUCTR, despite having a smaller number of depression trials (351 in total), it featured more trials centered on specific drugs. Notably, duloxetine was present in 14 trials, psilocybin in 10 trials, and ketamine in 8 trials. Other drugs, such as tocilizumab (1 trial), erythropoietin (3 trials), oxytocin (3 trials), dronabinol (1 trial), and sitagliptin (1 trial), though less represented, were still evident among the top drugs in EUCTR. Interestingly, we also discovered an experimental drug, TAK-653, an AMPA receptor positive allosteric modulator, specifically designed for treatment-resistant depression, present in one trial in the EUCTR [60]. Additionally, the novel experimental drug JNJ- 54175446, a selective purine P2X7 receptor antagonist, appeared in the ISRCTN but was found in only one trial [61].

**Fig. 4:**
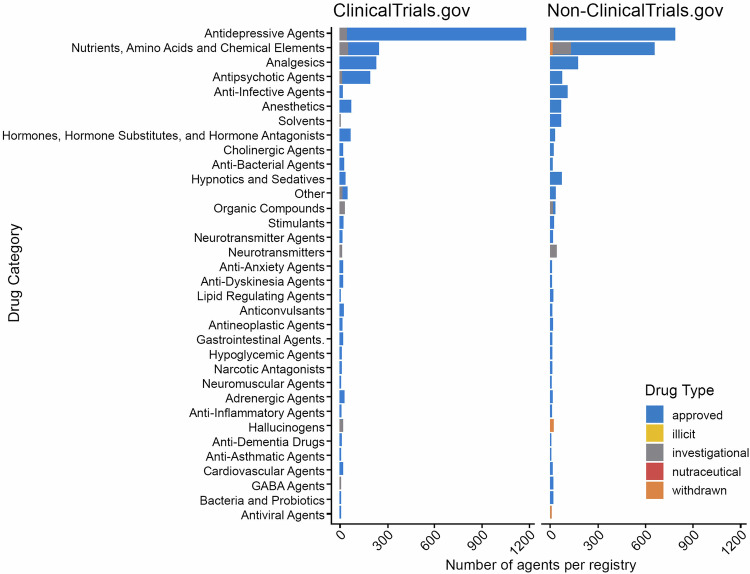
Drug categories in the ClinicalTrials.gov and non-ClinicalTrials.gov groups. The figure illustrates the proportion of drug categories per registry, focusing on depression trials with an interventional study design that involved drug treatment. In total, there were 3058 such trials analyzed. Antidepressive agents emerged as the most prevalent drug category, with a total of 1172 agents in ClinicalTrials.gov and 659 agents in the non-ClinicalTrials.gov group. Interestingly, less than 5% of antidepressive agents were investigational, with the majority already approved for use. Notably, most investigational drugs were found in the nutrients, amino acids, and chemical elements category, which was more abundant in the non-ClinicalTrials.gov databases (over 600 agents compared to 250 agents in the ClinicalTrials.gov group). The third most common category was analgesics, comprising entirely approved drugs. In the ClinicalTrials.gov database, antipsychotic agents occupied the fourth position, whereas in the non-ClinicalTrials.gov databases, the fourth spot was taken by anti-infective agents. In general, approved agents significantly outnumbered investigational ones in all categories. However, it’s worth noting that there were specific categories with a very small number of agents that were almost exclusively investigational. For instance, in the ClinicalTrials.gov database, categories like solvents, hallucinogens, and GABA agents fell into this group, whereas in the non-ClinicalTrials.gov group, the neurotransmitters category was entirely composed of investigational agents. These findings suggest a predominant focus on approved agents in depression trials.

To further analyze trends in pharmacotherapy for depression, we compared both approved and investigational drugs. Figure 4 displays the number of unique drugs categorized in ClinicalTrials.gov and non-ClinicalTrials.gov databases. In our analysis of interventional trials, we identified a total of 663 unique agents. Among these, 74.5% were approved drugs, 23.2% were investigational drugs, 1.8% had been withdrawn, 0.3% were nutraceuticals, and only 0.2% were illicit substances. As anticipated, the largest category was the approved drug category, which was used in 4 420 instances (accounting for 90%). In contrast, investigational drugs were used 454 times, making up 9.2% of the total. Further delving into the drug types, we discovered 34 different categories. Both ClinicalTrials.gov and non-ClinicalTrials.gov databases showed that the largest categories were antidepressive agents (1172 agents in ClinicalTrials.gov and 659 in non-ClinicalTrials.gov) and nutrients, amino acids, and chemical elements (250 agents in ClinicalTrials.gov and 659 agents in non-ClinicalTrials.gov). These findings suggest that trials registered in non-ClinicalTrials.gov tend to take a more alternative approach to treatment, often incorporating dietary supplements and nutrients. However, both databases also displayed a focus on analgesics, antipsychotic agents, and anti-infective agents, with the ClinicalTrials.gov group slightly prevailing in these categories. Nevertheless, the number of trials in each category did not exceed 300.

In our in-depth analysis of investigational drugs, we found a total of 154 different compounds that have not yet been approved by the FDA for any disease. Out of these investigational drugs, 36.4% have been the subject of studies conducted in non-ClinicalTrials.gov, 41.6% were studied through trials registered in ClinicalTrials.gov, and 22.1% were studied in both registry types. Additionally, we identified 236 drugs that have already received FDA approval for various indications. Among these FDA-approved drugs, 217 have not yet been approved specifically for major depressive disorder. These 217 drugs were included in further investigations, alongside the aforementioned investigational drugs, to explore their potential as pharmacotherapy for depression.

**Fig. 5:**
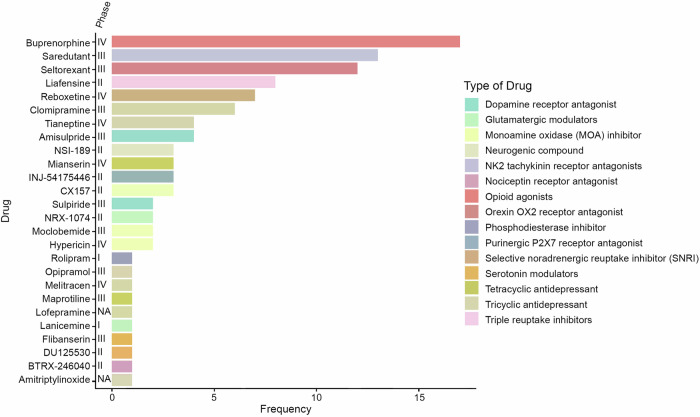
Investigational drugs in depression treatment. During the analysis of investigational antidepressive agents, several drugs emerged as the most common candidates for study. The indicated phases represent the highest phases that these drugs have reached in clinical trials. The frequency of each drug corresponds to the number of times the drug was mentioned in clinical trials. Among these investigational drugs, several reached the highest phase of 4. These drugs include buprenorphine, reboxetine, tianeptine, mianserin, hypericin, and melitracen. Two drugs, lanicemine and rolipram, were in phase 1 of clinical trials, indicating early-stage research. There were also two drugs for which phase division was not applicable (NA): lofepramine and amitriptylinoxide. Notably, buprenorphine was the only drug with more than 15 trials dedicated to its study. Interestingly, among the top investigational agents for depression treatment, no antibiotics or NSAIDs (non-steroidal anti-inflammatory drugs) were found, highlighting the focus on other types of compounds and therapies in trials.

Figure 5 presents the filtered data of FDA-approved and non-approved investigational antidepressive agents used in trials studying major depression. A total of 26 investigational antidepressive agents were identified, targeting 16 different drug targets. The findings indicate a diverse array of novel drug treatments being introduced, with the highest frequency observed for buprenorphine (opioid agonists), saredutant (NK2 tachykinin receptor antagonists), and seltorexant (orexin OX2 receptor antagonist). Out of the 26 identified drugs, 6 have reached the highest phase of 4 in their development (buprenorphine, reboxetine, tianeptine, mianserin, hypericin, and melitracen). It’s worth noting that the overall number of trials dedicated to these investigational drugs is relatively small, with only buprenorphine being present in more than 15 trials. While the number of trials may be limited, these results showcase promising new investigational drugs with diverse targets. The broad range of different drug targets includes traditional targets like dopamine receptor agonists and antagonists, glutamatergic modulators, MAO inhibitors, serotonin modulators, SNRIs, and Tricyclic and Tetracyclic antidepressants, alongside atypical drugs.

Table 1 lists FDA and EMA approved drugs for depression, comprising a total of 40 drugs obtained through extensive literature and database searches. The majority of these drugs fall into specific categories, including Serotonin and/or Norepinephrine Reuptake Inhibitors (12 drugs), Tricyclic Antidepressants (8 drugs), Atypical Antipsychotics (7 drugs), and Monoamine Oxidase Inhibitors (4 drugs). Notably, we also highlight some intriguing drugs, such as agomelatine, which acts as a melatonin agonist but was unfortunately discontinued by the FDA in 2011. Two noteworthy drugs in the table act as GABA (A) receptor positive allosteric modulators: brexanolone (FDA approved in 2019) and zuranolone (FDA approved in 2023). Both are designed for postpartum depression treatment and are under investigation for the treatment of major depressive disorder. Brexanolone is also being studied for super-refractory status epilepticus. NMDA receptor antagonists emerge as novel treatments, with esketamine gaining

**Table 1:** FDA and EMA-approved drugs for depression treatment.

Drug name	Pharmacol ogic classes	Target	EMA approval	FDA approval	Depression-related indications	Other indications
Agomelatine	Melatonin agonist	MT1, MT2 agonist; 5- HT2C antagon ist	2009	Discontinued in 2011	MDD (with sleep disorder)	–
Amitriptyline	Tricyclic antidepressant	NET, SERT Inhibitor	2017	1961	MDD (endogenous, psychotic, neurotic)	Neuropathic pain; Chronic tension-type headache; Migraine; nocturnal enuresis
Amoxapine	Tricyclic antidepressant	NET, SERT Inhibitor	2013	1992	MDD (neurotic, reactive, endogenous, psychotic)	–
Aripiprazole	Atypical antipsychotic	5-HT1A, D2 partial agonist; 5- HT2A antagonist	2004	2002	MDD (psychotic)	Bipolar I disorder; Schizophrenia
Brexanolone	Neuroactive steroid gamma-aminobutyric acid a receptor-positive modulator	GABA(A) positive allosteric modulator		2019	PPD	–
Brexpiprazole	Atypical antipsychotic	5-HT1A, D2 partial agonist; 5-HT2A antagonist	2018	2015	Adjunctive therapy in MDD	Adjunctive therapy in MDD; Schizophrenia; Agitation in Alzheimer’s disease
Bupropion	Aminoketone	NET, SERT Inhibitor	2002	1985	MDD (atypical, bipolar depression)	SAD; Smoking cessation; obesity
Citalopram	Serotonin reuptake inhibitor	SERT Inhibitor		1998	MDD	–
Clomipramine	Tricyclic antidepressant	NET, SERT Inhibitor	1998	1989	Depression with obsessive- compulsive component	OCD
Cariprazine	Atypical antipsychotic	5-HT1A, D2, D3 partial agonist; 5-HT2A antagonist	2017	2015 (Initial) 2022 (MDD)	Bipolar depression; Adjunctive therapy in MDD	Schizophrenia; Bipolar mania;
Desipramine	Tricyclic antidepressant	NET Inhibitor		1964	MDD (endogenous)	Neuropathic pain; Anxiety disorders
Desvenlafaxine	Serotonin and norepinephrine reuptake inhibitor	NET, SERT Inhibitor	2009	2008	MDD	Hot flashes
Dextromethorphan/Bu propion (Auvelity)	NMDA receptor antagonist	NMDA receptor antagonist	Not approved	2022	MDD	–
Doxepin	Tricyclic antidepressant	NET, SERT Inhibitor		1969	MDD (psychotic, manic-depressive, involutional, anxious)	Insomnia
Duloxetine	Serotonin and norepinephrine reuptake inhibitor	NET, SERT Inhibitor	2004	2004	MDD	GAD; Diabetic Peripheral Neuropathy; fibromyalgia; chronic musculoske letal pain
Escitalopram	Serotonin reuptake inhibitor	SERT Inhibitor	2010	2002	MDD (including acute treatment)	GAD
Esketamine	NMDA receptor antagonist	NMDA receptor antagonist	2019	2019	TRD	–
Fluoxetine	Serotonin reuptake inhibitor	SERT inhibitor	2006	1987	MDD (endogenous); TRD; Bipolar depression (+olanzapine);	PMDD
Fluvoxamine	Serotonin reuptake inhibitor	SERT inhibitor	2002	1994	MDD (endogenous)	Bulimia nervosa; OCD
Imipramine	Tricyclic antidepressant	NET, SERT inhibitor		1959	MDD (endogenous, psychotic, atypical)	Childhood enuresis
Isocarboxazid	Monoamine oxidase inhibitor	MAO – A, MAO – B inhibitor		1998	TRD (in case other drugs fail)	PD
Lumateperone	Atypical antipsychotic	SERT Inhibitor, D2 partial agonist, 5-HT2A antagonist		2019	Bipolar depression	Schizophrenia
Lurasidone	Atypical antipsychotic	D2, 5-HT2A antagonist	2014	2010 – schizophrenia 2013 - bipolar depression in adults 2018 - bipolar depression in pediatric patients	Bipolar depression	Schizophrenia
Levomilnacipran	Serotonin and norepinephrine reuptake inhibitor	NET, SERT inhibitor		2013	MDD	–
Milnacipran	Serotonin and norepinephrine reuptake inhibitor	NET, SERT inhibitor	Refused in 2009	2009	Short-term MDD symptoms relief	Fibromyalgia
Mirtazapine	Tetracyclic antidepressants	5- HT2A, 5-HT3, α1 antagonist	2008	1996	MDD (poststroke depression)	–
Nortriptyline	Tricyclic antidepressant	NET, SERT inhibitor		1964	MDD (endogenous, melancholic)	–
Olanzapine	Atypical antipsychotic	D2, 5- HT2A antagonist	1996	1996	bipolar depression; TRD	Schizophre nia; Bipolar I disorder
Paroxetine	Serotonin reuptake inhibitor	SERT inhibitor	2005	1992	MDD (endogenous)	GAD; OCD; PTSD; SAD; PMDD; PD IBS; Premature Ejaculation ; Menopause Vasomotor Symptoms
Phenelzine	Monoamine oxidase inhibitor	MAO – A, MAO – B inhibitor		1961	MDD (atypical, nonendogenous, neurotic)	–
Quetiapine	Atypical antipsychotic	D2, 5- HT2A antagonist, NET inhibitor	2014	2007	Bipolar Depression; Adjunctive therapy in MDD	Schizophrenia; Bipolar I disorder
Selegiline	Monoamine oxidase inhibitor	MAO - B inhibitor	2010	1989 – Parkinson 2006 - MDD	MDD (transderma l system)	Parkinson’s disease;
Sertraline	Serotonin reuptake inhibitor	SERT inhibitor	2009	1991	MDD (atypical, with cognitive improvement)	OCD; PTSD; SAD; PD
Tranylcypromine	Monoamine oxidase inhibitor	MAO – A, MAO – B inhibitor	2000 – UK (Parnate) 2013 – UK (Tranylcypro mine)	1961	TRD	–
Trazodone	Serotonin reuptake inhibitor	SERT inhibitor; 5- HT2A, 5- HT1A antagonist; 5- HT2C agonist		1981	MDD (endogenous)	–
Trimipramine	Tricyclic antidepressant	NET, SERT inhibitor	1966	1982	MDD (endogenous, neurotic)	–
Venlafaxine	Serotonin and norepineph rine reuptake inhibitor	NET, SERT inhibitor	2008	1993	MDD (refractory, endogenous)	GAD; SAD; PD
Vilazodone	Serotonin Modulators	SERT Inhibitor; 5HT1A partial agonist	Not approved	2011	MDD	–
Vortioxetine	Serotonin modulators	SERT Inhibitor; 5-HT1B partial agonist; 5-HT1A agonist; 5-HT3, 5-HT1D, 5-HT7 antagon ist	2013	2013	MDD	–
Zuranolone	Neuroactive steroid gamma-aminobutyric acid A receptor positive modulator	GABA(A) positive allosteric modulator	Not approved	2023	PPD	–

*MT* melatonin receptor, *5-HT* 5-hydroxytryptamine, *NET* norepinephrine transporter, *SERT* serotonin reuptake transporter, *GABA* gamma-aminobutyric acid, *NMDA* N-methyl- D-aspartate, *MAO* monoamine oxidase, *MDD* major depressive disorder, *SAD* seasonal affective disorder, *OCD* obsessive–compulsive disorder, *PMDD* Premenstrual dysphoric disorder, *GAD* Generalized anxiety disorder, *TRD* treatment-resistant depression, *PTSD* post-traumatic stress disorder, *PD* panic disorder, *PPD* postpartum depression.

FDA approval for treatment-resistant depression in 2019. In 2022, the FDA approved a novel formulation that includes an NMDA receptor antagonist and an aminoketone, specifically dextromethorphan/bupropion (marketed as Auvelity), for the treatment of Major Depressive Disorder. Additionally, in the same year, cariprazine was approved as an adjunctive treatment for depression. Our search revealed that only five drugs have received approval for treatment-resistant depression: fluoxetine, esketamine, isocarboxazid, tranylcypromine, and olanzapine. For each drug in Table 1, we made efforts to specify the depression subtypes in which they exhibit the most effectiveness, based on a thorough analysis of the literature. One noteworthy example is mirtazapine, which has demonstrated particular success in treating poststroke depression.

## Discussion

### Geographical distribution of clinical trials

We conducted an extensive analysis of depression trials, studying a total of 10,606 trials. While our research does show a growing presence of trials within ClinicalTrials.gov, a closer look at the annual trends reveals an interesting shift (as illustrated in Fig. 1). The ClinicalTrials.gov database experienced a sudden and noteworthy increase in registrations in the year 2005. This surge can be attributed to the 2005 policy established by the International Committee of Medical Journal Editors (ICMJE), which made trial registration a prerequisite for publication consideration [62]. That said, from 2005 to 2020, the proportion of studies registered outside of ClinicalTrials.gov also exhibited a remarkable rise. This trend reached a point where by 2018, there were more studies registered outside of ClinicalTrials.gov (501 trials in the non-CT group) than within it (484 trials in the CT group).

Among the prominent contributors outside of ClinicalTrials.gov, several registries stood out, including the Australian New Zealand Clinical Trial Registry (ANZCTR, 1031 trials), the Iranian Registry of Clinical Trials (IRCT, 576 trials), and the Chinese Clinical Trial Registry (CHiCTR, 489 trials). The ANZCTR database was established as a national research infrastructure in 2005, significantly boosting trial registration [63]. Despite generally positive feedback, including a rise in patient-reported outcomes and enhanced prospective registration, we observed that ANZCTR was underrepresented in the study of drug trends for specific diseases [64–67]. This issue was also partially noted with the IRCT database, although the reviews we did find primarily focused on phyto-drugs and phyto-solutions [68–70]. CHiCTR experienced the most substantial growth between 2016 and 2020, emerging as the largest non- ClinicalTrials.gov registry for depression trials in 2020. This surge in activity was paralleled by increased investments from Asian countries, particularly China, which tripled its funding for medical research education and personnel from $2.6 billion in 2004 to $9.7 billion in 2012 [71]. Furthermore, we observed a rising number of clinical trial reviews utilizing the CHiCTR database as their primary data source, partly due to increased research related to COVID-19 [72–75].

### Drug distribution in the ICTRP database

We conducted a comprehensive analysis of drugs studied within both the ClinicalTrials.gov and non-ClinicalTrials.gov groups using a word-matching algorithm, as depicted in Figs. 2 and 3. Figure 2 illustrates that both groups exhibited a similar pattern in the most frequently studied agents. These included ketamine, sertraline, duloxetine, and fluoxetine, respectively. Notably, among these top drugs, ketamine stands out as an atypical antidepressant, while the others belong to the SSRI (sertraline, fluoxetine) or SNRI (duloxetine) categories [6, 76]. Currently, ketamine is being explored as an adjunctive treatment for treatment-resistant depression [77]. Moreover, ketamine administered via intravenous infusion has shown positive effects in reducing suicidal ideation, mitigating PTSD severity, and generally improving conditions associated with heightened anxiety [78–81]. The persistence of the trend in ketamine trials may be attributed to the need for further investigation into its potential long-term side effects and the precise identification of depression subtypes where ketamine is most beneficial [77]. Based on our findings and a thorough literature review, traditional antidepressants such as SSRIs and SNRIs continue to maintain their popularity in clinical trials. This enduring preference can be attributed to several factors, including their established clinical efficacy and the ongoing exploration of novel effects, including their potential anti-inflammatory and antioxidant properties [43, 82–85].

Following an extensive examination of the top databases within the non-ClinicalTrials.gov group, we observed a notably distinct distribution of drugs within each individual registry, as depicted in Fig. 3. To illustrate, ketamine emerged as the top drug exclusively in the ANZCTR. Meanwhile, in the ISRCTN and EUCTR databases, agomelatine and psilocybin ranked among the top three agents, respectively. Psilocybin, a relatively novel compound primarily acting as a serotonin receptor agonist and previously recognized for its psychedelic properties, is now being repurposed for the treatment of conditions such as major depressive disorder, anxiety, and substance use [86–89]. Recent phase 2 clinical trials have reported a dose-dependent reduction in the Montgomery–Asberg Depression Rating Scale (MADRS) score following a three-week course of psilocybin [90]. However, it’s important to note that these trials, along with many others, have also reported a high incidence of side effects (up to 77%) and minimal impact on suicidal ideation. Consequently, there are a growing number of clinical trials adjusting the dose and potentially the drug formulation to optimize its therapeutic effects [90–94]. Interestingly, despite the substantial attention given to gut microbiota in the development of depression, we observed that *L. helveticus* (present in three trials) and *L. acidophilus* (present in one trial) were exclusively featured in the top drugs within the IRCT database. Notably, a recent review highlights the prevalence of probiotic studies within the ICTRP database, even when compared to ClinicalTrials.gov (as of 2020, ClinicalTrials.gov had 1 341 trials on probiotics versus 1 619 trials in the ICTRP) [49]. Based on our findings, we can propose that the IRCT database plays a significant role in contributing to the number of probiotic trials [95–97].

### Approved and investigational drugs

Out of all depression trials, 3058 interventional studies focused on drug treatments, encompassing 663 unique drugs, including neurotransmitters, amino acids, and organic compounds (Fig. 4). Most of these drugs were expectedly antidepressants. An analysis of registry groups revealed that non-ClinicalTrials.gov studies favored non-traditional nutrient-based treatments, while ClinicalTrials.gov studies leaned towards conventional antidepressant agents. This suggests that non-U.S. funded studies explore nutritional approaches more often. These nutritional compounds, such as omega-3 fatty acids, vitamin D, and magnesium, have been previously linked to major depression [98–100]. Regarding pharmacotherapy in depression trials, most drugs used were FDA-approved (76.8%), as there is an array of approved antidepressants available [101]. However, the challenge remains, as up to 30% of depressed patients do not respond to these drugs [102]. Identifying novel treatments like lanicemine, designed for treatment-resistant depression, becomes crucial. But our analysis showed lanicemine’s limited use in clinical trials (less than five instances), with controversial results suggesting modest improvements [103–105].

We identified 26 investigational antidepressant drugs by combining non-FDA approved with FDA-approved drugs. These drugs target a variety of receptors, including well-known ones like glutamatergic, serotonin, and dopamine modulators, as well as unconventional targets (Fig. 5). Among the most frequent drug types were opioid agonists, orexin OX2 receptor antagonists, NK2 tachykinin receptor antagonists, and triple reuptake inhibitors. Notably, buprenorphine, originally used for opioid use disorders, emerged as the most common drug, showing antidepressant effects through kappa opioid receptor antagonism [106]. Recently, buprenorphine is being combined with several other drugs in clinical trials, including naloxone and samidorphan [107, 108]. Despite being well-tolerated, these combinations presently show only moderate efficacy. Saredutant, an NK2 receptor antagonist, was also among the most frequently studied investigational drugs. While previous attempts with MK-869 (aprepitant) targeting the same receptor were unsuccessful, saredutant displayed more promising results in animal models and remains under investigation [109, 110]. However, a recent review indicated new potential for aprepitant due to its ability to promote wound healing in diabetic patients as well as having some antidepressive properties [111]. Seltorexant, an orexin OX2 receptor antagonist, was also frequently studied. Research in rodents and humans suggests its potential role in depression’s pathogenesis by influencing hypothalamic cells expressing high orexin concentrations [112].

The analysis of approved drugs highlights the emergence of pharmacological classes such as GABA modulators and NMDA antagonists. In the GABA modulator class, both brexanolone (2019) and zuranolone (2023) have received FDA approval exclusively for the treatment of postpartum depression. On the other hand, NMDA antagonists like dextromethorphan/bupropion (2022) and esketamine (2019) can be used for the treatment of Major Depressive Disorder (MDD) as well as its treatment-resistant subtypes, as shown in Table 1. GABA modulators represent a significant departure from the traditional serotonin-norepinephrine-dopamine depression hypothesis. They offer a novel pharmacological action and are the first available drug options specifically for patients with postpartum depression [113–115]. Zuranolone and the investigational drug ganaxolone are also being tested in the treatment of MDD, with remission achieved in nearly half of the subjects [116]. In contrast, esketamine operates by inhibiting NMDA receptors, which have a physiological action opposite to GABA [117, 118]. This approach has shown promising results, particularly in the treatment of treatment-resistant depression (TRD) [119]. In a recent study, it was observed that by week 8, remission was achieved in 27.1% of enrolled patients with TRD who received esketamine, while only 17.6% of patients taking quetiapine achieved remission during the same timeframe [120]. Despite the emergence of these new therapeutic options, the majority of proposed depression treatments are either investigational or experimental. This underscores the need for further clinical trials, with a focus not only on standard depression treatment options.

## Conclusion

The ICTRP database is a comprehensive platform, offering a wide range of clinical trial information, not limited to ClinicalTrials.gov. Our approach is based on [56]/our previous analysis where we use a novel method to uncover hidden duplicates during the ICTRP analysis. Among the top databases in the non-ClinicalTrials.gov group, the ANZCTR (1031 trials), ISRCTN (501 trials), IRCT (576 trials), and CHiCTR (489 trials) stood out. CHiCTR notably showed a significant increase, registering 160 trials in 2020. When dividing the data into just two groups, ClinicalTrials.gov and non-ClinicalTrials.gov, the drug distribution remained fairly consistent, with ketamine, sertraline, fluoxetine, and duloxetine ranking high in both. However, when examined individually, each registry showcased a unique drug landscape. For instance, IRCT notably features probiotics (*L. helveticus* and *L. acidophilus*), ANZCTR focuses heavily on ketamine, minocycline is exclusively present in IRCT and EUCTR databases, and psilocybin was represented more often in the EUCTR. These findings underscore the substantial diversity among global databases, suggesting researchers should not restrict their searches solely to ClinicalTrials.gov. Categorizing studies by different drug classes highlights that ClinicalTrials.gov tends to favor traditional antidepressants, while non-ClinicalTrials.gov studies lean toward atypical nutritional supplements. In both cases, less than 5% of studied agents are investigational, with the majority being approved drugs. We identified 663 unique agents for depression that include supplementary data for further analyses. Our investigation of interventions highlights novel drug targets, with the most common investigational antidepressants acting on opioid receptors, NK2 tachykinin receptors, and orexin OX2 receptors. These targets offer new prospects for treating drug-resistant depression.
